# Spinal Intramedullary Tuberculosis

**DOI:** 10.1055/s-0037-1599823

**Published:** 2017-03-30

**Authors:** Prithvi Varghese, Muhammed Jasim Abdul Jalal, Julio Chacko Kandathil, Iona Leekha Mathew

**Affiliations:** 1 Department of Neurosurgery, VPS Lakeshore Hospital, Kochi, Kerala, India; 2 Department of Internal Medicine and Rheumatology, VPS Lakeshore Hospital, Kochi, Kerala, India; 3 Department of Radiology, VPS Lakeshore Hospital, Kochi, Kerala, India; 4 Department of Pathology, VPS Lakeshore Hospital, Kochi, Kerala, India

**Keywords:** tuberculosis, spinal, intramedullary, tuberculoma

## Abstract

Tuberculosis of the central nervous system accounts for approximately 1% of all cases of tuberculosis and 50% of these involve the spine. Intramedullary involvement is rare in tuberculosis. Clinical presentation of spinal intramedullary tuberculosis (SIMT) is similar to intramedullary spinal cord tumor. Here, we report the case of a 49-year-old female with dull aching pain of both upper limbs of 1-week duration. On examination, she had no motor deficits. All the deep tendon reflexes were normal. The plantar responses were flexor bilaterally. Cervical spine imaging favored intramedullary tumor. She had partial relief of symptoms with steroid treatment. Repeat imaging done 1 month later revealed mild interval enlargement of the intramedullary lesions and multiple enlarged mediastinal and hilar nodes. Endoscopic ultrasound-guided fine-needle aspiration cytology of mediastinal nodes was suggestive of granulomatous inflammation. Hence, SIMT was considered as the probable diagnosis. The patient was started on antituberculosis therapy.

According to the World Health Organization statistics for 2011, out of the estimated global annual incidence of 8.7 million tuberculosis (TB) cases, 2.2 million were from India alone. TB of the central nervous system (CNS) accounts for approximately 1% of all cases of TB and 50% of these involve the spine. 1 2 Spinal TB presents as various types of lesions.

Pott's disease of the spine (tuberculous spondylitis) is the most common (60%), followed by arachnoiditis (20%), meningitis (12%), and intramedullary lesion (8%). Meningitis is the most common form of spinal intradural TB. Intramedullary involvement is rare in TB and usually present in the form of radiculomyelitis, transverse myelitis, intraspinal granulomas, or thrombosis of anterior spinal artery. 3 Clinical presentation of spinal intramedullary TB (SIMT) is similar to intramedullary spinal cord tumor. When diagnosed in time and managed appropriately, SIMT has good prognosis.

## Case Report

A 49-year-old Indian female presented with dull aching pain of both upper limbs of 1-week duration. The patient also had episodes of difficulty in moving both upper limbs. There was no significant past illness. General examination was normal. The patient was afebrile with a pulse rate of 82 per minute and blood pressure of 140/80 mm Hg. She was conscious and oriented. PEARL [Pupils equal and reacting to light]. There were no motor deficits. All the deep tendon reflexes were normal. The plantar responses were flexor bilaterally. All other systems were within normal limits.

**Fig. 1:**
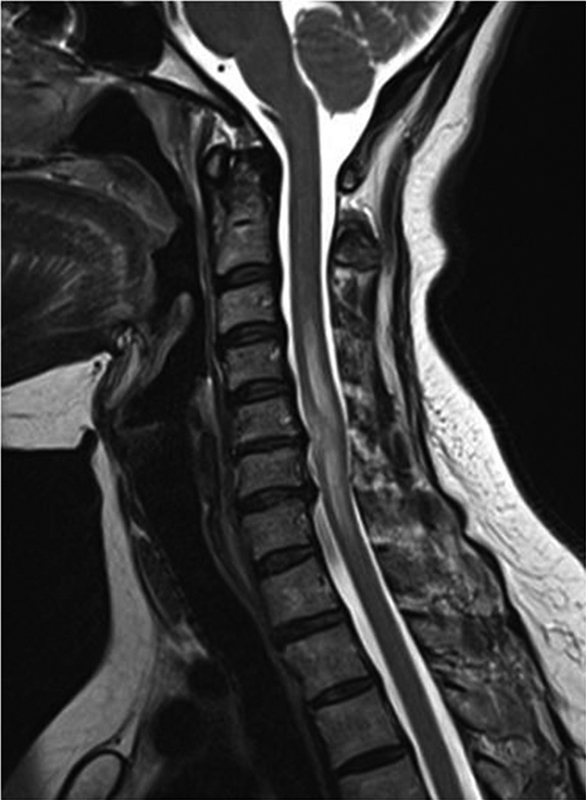
Cervical spine sag T2-weighted image showing swollen cervical cord with T2-weighted high-signal edema extending from C3 to C7 levels.

**Fig. 2:**
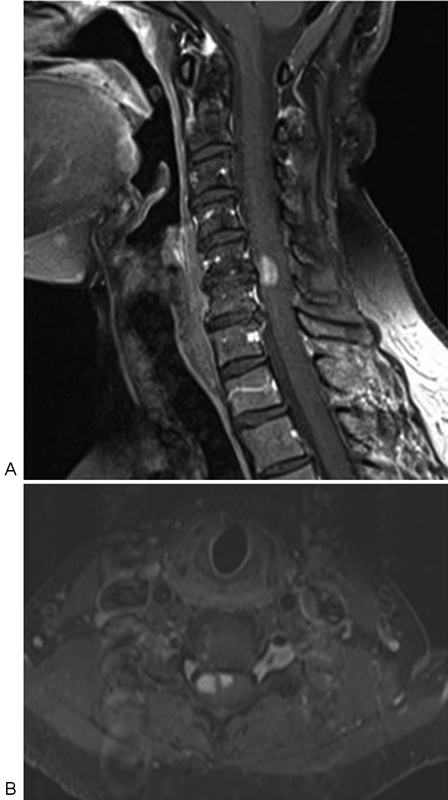
( **A** ) Cervical spine sagittal and ( **B** ) axial postcontrast T1-weighted fat suppressed images showing enhancing intramedullary cord lesions at C5–C6 level situated in the anterior midline and right anterolateral aspect.

Hemogram, liver function, renal function, and coagulation profile were all within normal limits. Magnetic resonance imaging (MRI) of cervical spine ( Figs. 1 and 2A, B ) showed cord edema and swelling from C3 to C7 levels with two enhancing intramedullary cord lesions in anterior and right anterolateral aspect of cervical cord at C5–C6 level measuring 0.4 × 0.4 × 0.8 cm and 0.6 × 0.7 × 1.1 cm, respectively. Cerebrospinal fluid (CSF) study was inconclusive (CSF was clear; CSF pressure: 120 mm H _2_ O; CSF sugar: 48 mg/dL; CSF protein: 21 mg/dL; 5 cells/mm ^3^ with occasional lymphocytes; and CSF culture was negative for pus cells, bacteria, and acid fast bacilli). At this stage, the possibility of an intramedullary tumor was considered. However, since the symptoms were rather acute in presentation, patient was treated with a course of steroids (intravenous methylprednisolone, 1 g, once daily for 5 days).

**Fig. 3:**
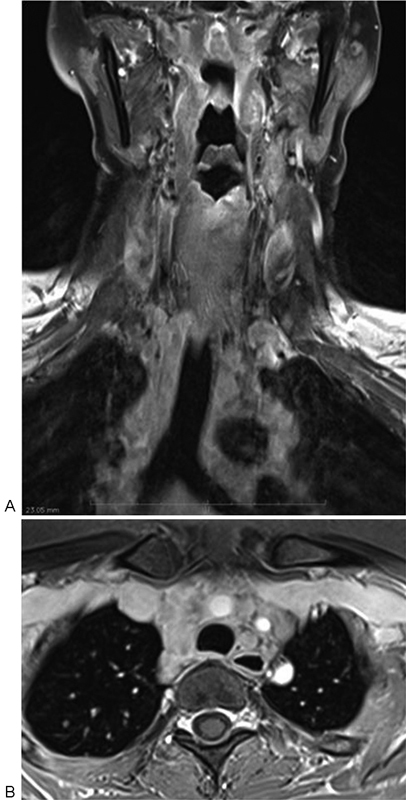
( **A** ) Coronal T1-weighted fat suppressed and ( **B** ) axial three-dimensional volumetric interpolated breath-hold examination postcontrast images through mediastinum showing extensive mediastinal and hilar adenopathy.

Patient had partial relief of symptoms with steroid treatment. Repeat MRI done 1 month later revealed mild interval enlargement of the intramedullary lesions. However, the repeat MRI study also showed multiple enlarged mediastinal and hilar nodes ( Fig. 3A, B ). Subsequent computed tomography of the thorax revealed extensive mediastinal and hilar adenopathy with no parenchymal lung lesion.

**Fig. 4:**
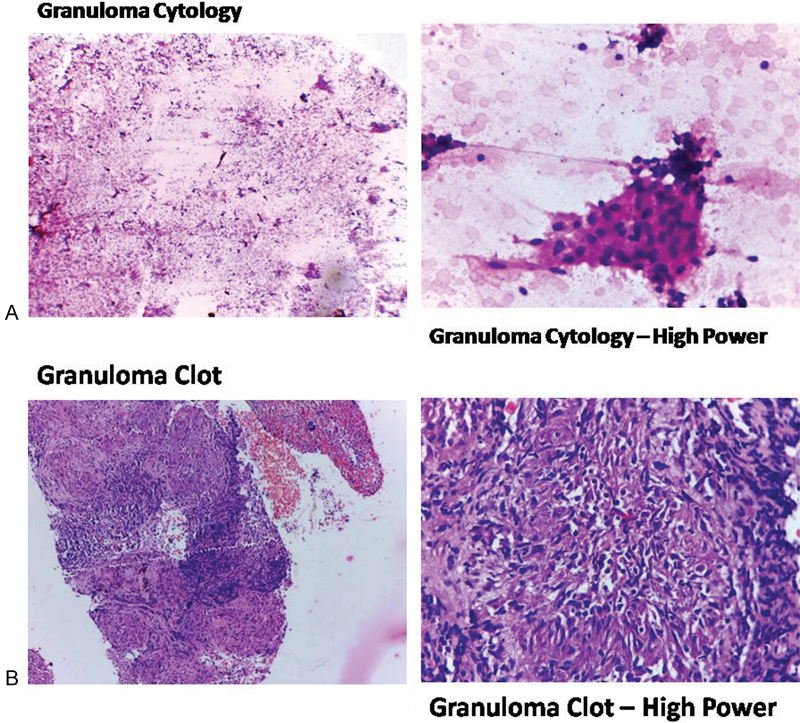
( **A** ) Granuloma cytology. ( **B** ) Granuloma clot.

Endoscopic ultrasound-guided fine-needle aspiration cytology of mediastinal nodes showed several epithelial cell granulomas and lymphoid cells suggestive of granulomatous inflammation with suspicious small foci of necrosis ( Fig. 4A, B ). Hence, SIMT was considered as the probable diagnosis. The patient was started on anti-TB therapy with isoniazid, rifampicin, pyrazinamide, and ethambutol.

**Fig. 5:**
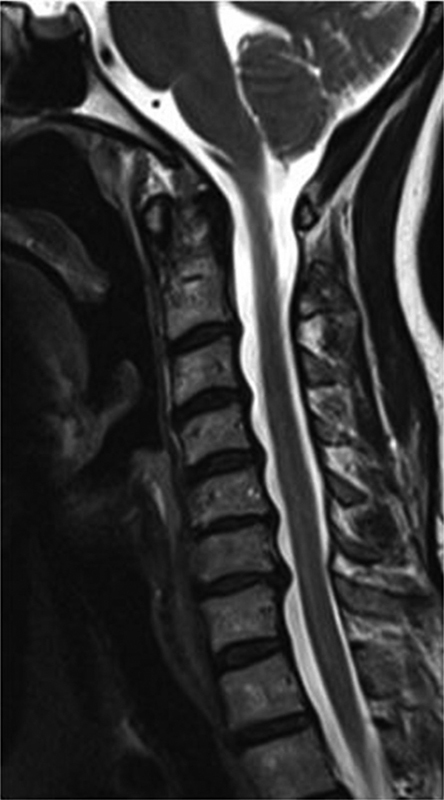
Cervical spine sag T2-weighted image postantituberculous therapy showing resolution of cord edema.

**Fig. 6:**
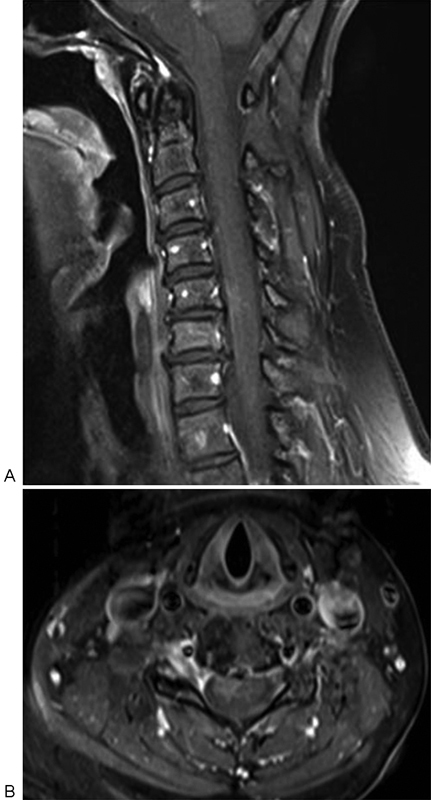
( **A** ) Cervical spine sagittal and ( **B** ) axial post contrast T1-weighted fat suppressed images postantituberculous therapy showing ill-defined residual enhancement at the site of C5–C6 level intramedullary lesions.

Repeat MRI of cervical spine ( Figs. 5 and 6A, B ) after 3 months of ATT showed resolution of the cervical intramedullary lesions and oedema. There was minimal diffuse residual enhancement at the C5-C6 level. The patient completed 7 months of ATT with isoniazid, rifampicin, pyrazinamide, and ethambutol. Rifampicin and isoniazid was continued for another 2 months. She had total relief of her symptoms following the ATT. A repeat MRI was not done due to financial reasons. On follow up at 2 years following ATT, she is asymptomatic and has no neurological deficits.

## Discussion

Spinal TB presents as various types of lesions. Pott's disease of the spine (tuberculous spondylitis) is the most common (60%), followed by arachnoiditis (20%), meningitis (12%), and intramedullary lesion (8%). 4 Dastur and Wadia described four major mechanisms that lead to spinal cord parenchymal involvement in TB 5 :

- Edema of border zones of the cord probably secondary to venous impediment due to pressure associated with meningitis.
- Ischemic myelomalacia resulting from vasculitis or postthrombotic occlusion of meningeal vessels.
- Infarction of the cord from vascular occlusion.
- Formation of intramedullary tuberculomas with pericentral necrosis.

SIMT was first reported by Abercrombie in 1828. 6 In 1960, Arseni and Samitca 7 reported 210 cases of CNS tuberculoma, of which only 9 were spinal masses. Linet al 8 9 compiled 105 cases of intramedullary tuberculomas in 1960. In 17 of these cases, the diagnosis was made surgically, but 88 cases were discovered at postmortem examinations.

The types of lesions that result from discharge of the bacilli into the CSF depend on the virulence of the bacteria and the immune resistance of the host. A study of immunological parameters showed a relation between the development of tuberculous meningitis in children and significantly lower count of CD4 T-lymphocytes when compared with children who had pulmonary complex only. 10

The brain lesion also originates from hematogenous spread from the lungs. When there is a sizeable inoculation with inadequate cell-mediated immunity, the parenchymal lesion may develop into a tuberculoma or a tuberculous abscess. 10

In Dastur's excellent review and summary of the tuberculoma series, 260 cases were in the brain and 6 were in the cord. Of the 74 tuberculous paraplegias in this group, 44 were extradural, 4 were subdural, 4 were combined subdural and extradural, and 6 were intramedullary. 11

SIMT is mostly induced by hematogenous dissemination or CSF infection. However, in a few cases, it is caused by local spreading of spinal TB. It is important to rule out pulmonary TB or extrapulmonary TB in patients with SIMT. SIMT most commonly involves the thoracic spinal cord (55%). 12 13 SIMT is also reported in patients with human immunodeficiency virus, autoimmune disease, especially systemic lupus erythematosus, and patients undergoing immunosuppressive treatment due to liver transplantation. 14 Patients frequently present with signs of spinal cord compression such as progressive lower limbs weakness, paresthesia, quadriplegia, paraplegia, and bladder and bowel dysfunction.

If diagnosed early, SIMT has good response to medical treatment. Anti-TB medications and a short course of injectable steroids offer an effective, inexpensive, safe, and feasible option for treating SIMT, especially in developing countries. 15 Short-term steroids may be particularly helpful to reduce the perilesional edema. Usually, the conservative treatment is successful in achieving complete clinical neurologic recovery over a period of 1 to 2 years, which is also accompanied by resolution of the tuberculomas. 16

Surgical intervention should be considered for cases showing progressive deficits in spite of adequate medical management and large lesions causing significant compression. With skilled microsurgical techniques, it is possible to safely excise the SIMT as these lesions are well circumscribed. MacDonnell et al have reported 65% recovery after surgical treatment. 16

Potential benefits of surgery were less kyphosis, immediate relief of compressed neural tissue, quicker relief of pain, higher percentage of bony fusion, quicker bony fusion, less relapse, earlier return to previous activities, and less bone loss. It may also prevent late neurologic problems due to kyphosis of the spine if fusion has not occurred.

Two types of surgical procedures are performed. One is debridement of the infected material. In this form of surgery, no attempt is made to stabilize the spine. The other procedure is debridement with stabilization of the spine (spinal reconstruction). This is a more extensive procedure, and the reconstructions are performed with bone grafts. Stabilization may also be done using artificial materials such as steel, carbon fiber, or titanium.

In 2007, Park et al conducted a retrospective study and analyzed the treatment outcome in patients with spinal TB. Of 116 patients, 47 (35%) had severe symptoms. Radical surgery was performed in 84 (62%) patients. Twenty patients were treated with short-term chemotherapy, whereas 96 underwent long-term antituberculous treatment. At the end of chemotherapy, 94 patients had achieved a favorable status and 22 had an unfavorable one. Age and radical surgery were significantly related to a favorable outcome by logistic analysis. 17

The prognosis for spinal TB is improved by early diagnosis and rapid intervention. A high degree of clinical suspicion is required if patients present with chronic back pain, even in the absence of neurologic symptoms and signs. Medical treatment is generally effective. Surgical intervention is necessary in advanced cases with marked bony involvement, abscess formation, or paraplegia. Spinal TB affects young people; therefore, efforts should be made for its effective prevention. Controlling the spread of TB is only way available to prevent spinal TB.
